# Enhanced Telehealth Home-Monitoring Intervention for Vulnerable and Frail Patients after Cardiac Surgery (THE-FACS Pilot Intervention Study)

**DOI:** 10.1186/s12877-022-03531-4

**Published:** 2022-11-05

**Authors:** Shreya Sarkar, Jeffrey MacLeod, Ansar Hassan, Keith R. Brunt, Krisan Palmer, Jean-François Légaré

**Affiliations:** 1 New Brunswick Heart Centre, 400 University Avenue, Saint John, PO Box 2100, NB E2L 4L2 Canada; 2 Dalhousie Medicine New Brunswick, Saint John, New Brunswick Canada; 3 Maine Medical Center, Portland, ME USA; 4 Department of Pharmacology, Dalhousie University, Halifax, NS Canada; 5 Horizon Virtual Care Program, Horizon Health Network, Saint John, New Brunswick Canada

**Keywords:** Technology, Outcomes, Hospital readmission, Feasibility, Follow-up, Frailty, Remote care, Digital health

## Abstract

**Background:**

Frail cardiac surgery patients have an increased risk of worse postoperative outcomes. The purpose of this study was to evaluate the implementation of a novel Telehealth Home monitoring Enhanced-Frailty And Cardiac Surgery (THE-FACS) intervention and determine its impact on clinical outcomes in frail patients post-cardiac surgery.

**Methods:**

Frail/vulnerable patients defined by Edmonton Frailty Scale (EFS > 4) undergoing cardiac surgery were prospectively enrolled (November 2019 -March 2020) at the New Brunswick Heart Centre. Exclusion criteria included age < 55 years, emergent status, minimally invasive surgery, lack of home support, and > 10-days postoperative hospital stay. Following standard training on THE-FACS, participants were sent home with a tablet device to answer questions about their health/recovery and measure blood pressure for 30-consecutive days. Transmitted data were monitored by trained cardiac surgery follow-up nurses. Patients were contacted only if the algorithm based on the patient’s self-collected data triggered an alert. Patients who completed the study were compared to historical controls. The primary outcome of interest was to determine the number of patients that could complete THE-FACS; secondary outcomes included participant/caregiver satisfaction and impact on hospital readmission.

**Results:**

We identified 86 eligible (EFS > 4), out of 254 patients scheduled for elective cardiac surgery during the study period (vulnerable: 34%). The patients who consented to participate in THE-FACS (64/86, 74%) had a mean age of 69.1 ± 6.4 years, 25% were female, 79.7% underwent isolated Coronary Artery Bypass Graft (CABG) and median EFS was 6 (5–8). 29/64 (45%) were excluded post-enrollment due to prolonged hospitalization (15/64) or requirement for hospital-to-hospital transfer (12/64). Of the remaining 35 patients, 21 completed the 30-day follow-up (completion rate:60%). Reasons for withdrawal (14/35, 40%) were mostly due to technical difficulties with the tablet. Hospital readmission, although non-significant, was reduced in THE-FACS participants compared to controls (0% vs. 14.3%). A satisfaction survey revealed > 90% satisfaction and ~ 67% willingness to re-use a home monitoring device.

**Conclusions:**

THE-FACS intervention can be used to successfully monitor vulnerable patients returning home post-cardiac surgery. However, a significant number of frail patients could not benefit from THE-FACS given prolonged hospitalization and technological challenges. Our findings suggest that despite overall excellent satisfaction in participants who completed THE-FACS, there remain major challenges for wide-scale implementation of technology-driven home monitoring programs as only 24% completed the study.

**Supplementary Information:**

The online version contains supplementary material available at 10.1186/s12877-022-03531-4.

## Introduction

It is estimated that > 10% of all cardiac surgeries take place in frail adults and this has been increasing [1]. Frailty has been shown to be an independent predictor of increased healthcare resource utilization [2]. Furthermore, frail patients discharged after cardiac surgery have an increased risk (up to 30%) of hospital readmission or emergency room visits [3]. However, there are currently no standard approaches to the care of patients with frailty and improve their ability to be home after cardiac surgery [1]. This is particularly relevant given that the Canadian population is aging at an increasing rate with almost 20% of Canadians aged > 65 years in 2020 (Statistics Canada, https://www150.statcan.gc.ca/n1/en/catalogue/91-520-X).

Smartphone and device technology has recently become increasingly attractive to allow remote monitoring of patients presenting with different health complications [4]. Not surprisingly, home monitoring of patients after discharge post-cardiac surgery is an emerging trend [5], with proposed advantages for both the patients and the health care system. The advantages of such an approach can be twofold: while patients and their caregivers can save on cost, time, and efforts for return clinic visits while remaining in contact with their healthcare team, the healthcare system can potentially save on both time and the availability of additional spots for new patients [4]. Home monitoring can be especially valuable for elderly patients, for whom traveling may be particularly onerous [6]. The use of telehealth in cardiac surgery patient care has increased exponentially, more so under the current Covid19 pandemic [7–10]. However, a limited number of studies have explored the utility of a technology-based home monitoring specifically for frail, elderly patients after cardiac surgery.

Telehealth technologies are not new to the New Brunswick Heart Center (NBHC). The NBHC has been recognized internationally since 1998 when it deployed the first of its kind post-cardiac surgery home monitoring program over the standard telephone line, which is what the majority of people had in their homes at that time. This consisted of daily real-time interactive audio and video, blood pressure, oxygen saturation, and live 3 lead electrocardiographic monitoring. Nurses could monitor patients from their own homes and dispatch information to receiving emergency departments in advance of the patient they were directing to them as necessary. The equipment has since been updated and is reserved for those patients most in need of virtual “hands-on” follow-up outside of the current standard of care. Currently, the standard at the NBHC remains a nurse-driven program that allows regular communication via telephone to all patients after heart surgery (once per week) [11]. In the present study, we wanted to test the feasibility and impact of using a novel electronic tablet-based system with software designed to engage patients to provide responses that were capable of identifying patients at risk, so that a care team can be notified. The study population chosen was patients deemed to be vulnerable or frail based on standardized Edmonton Frailty Scale (EFS) testing. The objective was to understand whether a technology-based home monitoring platform could be used to monitor patients based on self-reported data, and participant/ caregiver satisfaction with using the same.

## Materials and methods

### Study design and study population

The study is a prospective and a non-randomized case- control pilot study, designed based on the CONSORT 2010 guidelines but tailored according to the current study [12]. All consecutive patients who underwent non-emergent isolated coronary artery bypass grafting (CABG), isolated valve repair/replacement, or combined CABG/valve at the NBHC between November 2019 to March 2020 were screened using the EFS. Patients with an EFS > 4 were considered for inclusion in the study. Patients were excluded if they were less than 55 years of age, if they underwent a minimally invasive procedure, if they had pre-existing high-risk conditions such as dialysis and acute infective endocarditis, cognitive inabilities (cognitive deficits, visual impairments, inability to read, major difficulties with technology) or social barriers (lack of family or potential caregivers to provide support). Importantly, we also excluded patients with a prolonged hospitalization (length of stay, LOS > 10 days) or patients who were discharged to another institution for recovery to ensure that we focussed on early discharge home after surgery. Propensity score-matched historical controls were used for the comparison of outcomes. These patients were admitted and treated at the NBHC between 2005 to 2017.

### Research Ethics Board approval

The Research Ethics Boards (REB) of Horizon Health Network (REB File # 100,097) and the University of New Brunswick (REB File # 048–2019) approved the study before it commenced. Written, informed, and in-person consent was also obtained from the patients before they participated in the study. The research was performed in accordance with the Declaration of Helsinki, and the study was registered in ClinicalTrials.gov PRS, a publicly accessible primary register (NCT05349708, initial release 06/04/2022).

### Frailty assessment

All eligible patients were evaluated at baseline by a research coordinator (Supplementary Fig. 1). Screening for frailty was performed using the EFS, which determines frailty based on a 17-point scale, and a score > 4 is considered vulnerable. The use of the EFS in patients undergoing cardiac surgery has been well established [13]. Once identified patients were approached for consent to participate in our research study. Additional baseline frailty assessment also included assessment using the Clinical Frailty Scale (CFS) and the Katz index [14, 15]. Heat maps for frailty were generated using the median and quartiles to generate a frailty gradient (Q1 = fit, Q2 = mildly frail, Q3 = moderately frail, Q4 = severely frail).

Duke Activity Status Index (DASI) was scored on all patients to provide a functional assessment of the patients [16]. Physical measures of frailty were also collected for all patients. Specifically, the handgrip strength test, quantified by the amount of static force that a hand can squeeze around a dynamometer, is commonly used as an indicator of overall health and is negatively associated with frailty and heart diseases [17, 18]. For this, the patients were asked to exert maximal effort to grip the dynamometer, and 3 readings were recorded for each hand [18, 19]. Lower body strength was assessed using a 15-s chair stand test. The chair stand test is a measure of leg strength and endurance and the ability to perform the most demanding daily life activities [20]. A chair sit-stand is a reliable method of detecting cardiac function and frailty in older adults [20, 21]. In this test, the patients were asked to sit down and stand up on a specific chair as many times as possible within 15 s [20]. All tests were performed prior to surgery and heat maps were generated as mentioned previously.

### Telehealth enhanced home- monitoring (THE-FACS intervention)

The study was designed based on the framework of complex interventions, namely, identifying the intervention (THE-FACS), testing the feasibility and implementation and finally evaluating its benefit [22]. THE-FACS intervention, consisting of an electronic tablet and software provided by Medtronic (Medtronic Inc. ©) was given to patients who met the eligibility criteria for the study and were successfully discharged home within 10 days of their surgery (Supplementary Fig. 1). Standardized education was provided to patients and nominated caregivers (family members or friends) including training on the electronic interface. Established protocols were already in place to guide our nursing staff in the education of patients and their families in preparation for discharge (current Telehealth monitoring program). There are 5 nurses involved in the current Telehealth program and 5 surgeons who provided input in any optimization of the standardized protocols already in place as it related to the present study. In addition to standard hospital care, all materials necessary for the enhanced home monitoring were given to each patient at the time of discharge for use beginning the following morning. The intervention consisted of a tablet (electronic device) already enabled with cellular data and Wi-Fi, Bluetooth-paired with a blood pressure monitor to measure blood pressure and heart rate. Patients enrolled in the program submitted daily “Health Checks” through the application on the tablet called NetResponse. Daily Health Checks are based on Clinical Practice Guidelines and are designed to:

- ◦ ask symptom-based questions tailored to a patient who has just undergone heart surgery,
- ◦ use branching logic to ask more specific questions about patient answers that could raise concerns,
- ◦ deliver just-in-time education and self-management tips, also using branching logic to deliver the right content in response to a patient’s previous answers,
- ◦ collect vital signs via the blood pressure and heart rate monitor,

The novelty of the intervention involved the use of an electronic algorithm-driven process designed to filter responses and screen for patients at risk and notify the care team promptly. Notifications allowed Telehealth nurses to contact the patient and address the issue at hand. The approach was designed to focus on communication with patients in need rather than all patients, regardless of need. The enhanced home monitoring was in place for 30 consecutive days in all enrolled patients.

A single phone call was made by the Telehealth staff for each enrolled patient and was carried out immediately after the patient completed the first health check using the tablet. This allowed our staff to remind patients of the importance of their routine health checks and other routine healthcare follow-up (taking medications and contacting 911 if they have an emergency).

### Usability of THE-FACS intervention

After completing the study, all participants were called via telephone and asked to complete a satisfaction survey about the program, which consisted of 6 Likert-like questions [23]. The questions encompassed three domains- training on the use of THE-FACS intervention, ease of use and user satisfaction (Supplementary Table 1). The percentage of patients who responded to specific questions were plotted graphically to compute the rates of response for the participant satisfaction survey. This helped evaluate the feasibility of using THE-FACS intervention.

### Outcomes of interest and statistical analysis

Feasibility was assessed by our ability to enroll/ train patients and complete the study including a satisfaction survey. The clinical outcome of interest was hospital readmission within the 30 days of discharge in our intervention cohort compared to historical controls at a 2:1 ratio as described by others [24–26]. Only enrolled patients who completed the 30-day study (completed group) were compared with a 2:1 number of propensity score-matched historical controls from the same cardiac surgery population (control group). These groups were compared in terms of baseline and intraoperative characteristics as well as in-hospital and 30-day outcomes. All data were obtained from the NBHC Cardiac Surgery Registry, a detailed observational registry that includes all cardiac surgery encounters at the NBHC as previously described [27]. Categorical variables were reported as the number of observations and percentages. Continuous variables were summarized as mean (standard deviation) and median (interquartile range). Comparisons between groups were made using chi-square, Fisher’s exact test, student’s t-test, and Kruskal–Wallis test where appropriate. p-values < 0.05 were considered statistically significant. All data analysis was performed using GraphPad Prism 6 (GraphPad Software Inc, La Jolla, CA) and R version 3.6.3 (R Foundation for Statistical Computing, Vienna, Austria).

## Results

### Patient population enrolled

During the study period from November 2019 to March 2020, all 254 patients undergoing elective cardiac surgery were screened. Among them, 86 patients met our inclusion/ exclusion criteria. This shows that 33.9% of patients undergoing cardiac surgery can be defined as vulnerable or frail with a median EFS score of this population of 6 (IQR 5–8).

**Fig. 1:**
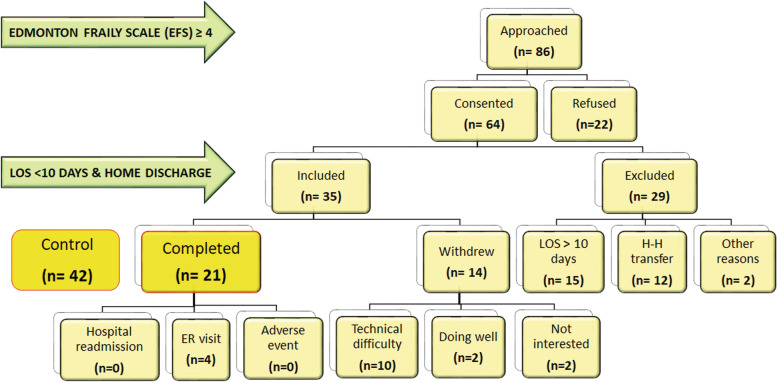
Patient flow during THE-FACS study. The distribution of patients who were approached for THE-FACS study, consented and were finally included according to inclusion and exclusion criteria. Patients who completed the study (yellow box) were compared to double the number of historical controls for comparison of outcomes. Abbreviations- H-H: hospital to hospital transfer; LOS: Length Of Stay; ER: Emergency Room

**Fig. 2:**
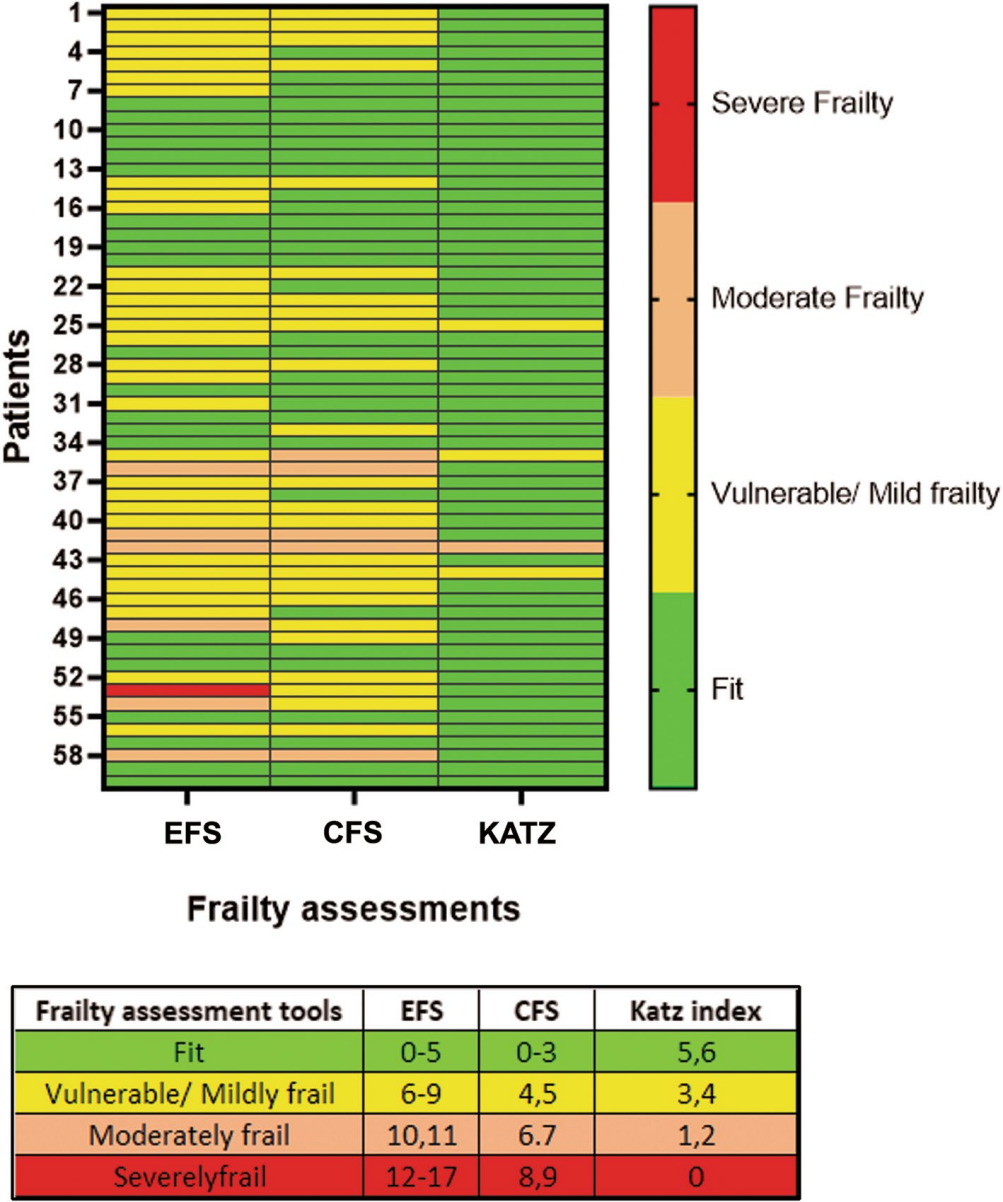
Heat map of frailty in patients who consented to THE-FACS study. A heat map was generated to compare frailty using Edmonton Frailty Scale (EFS), Clinical Frailty Scale (CFS), and Katz Index. Patients were categorized as fit (green), vulnerable/ mildly frail (yellow), moderately frail (pink), and severely frail (red). The table shows the score ranges used in the individual frailty assessment tools. Most patients who consented were fit to moderately frail

Out of the 86 patients approached, 64 patients (74.4%) consented to participate in the study (Fig. 1) and had the following characteristics (Table 1). The mean age of consented patients was 69 ± 7 years, 25% were female, 17.2% had a left ventricle ejection fraction (LVEF) of less than 40%, 35% required urgent surgery while in hospital and 11% underwent a combined procedure (Table 1). To represent individual patient frailty measurements, we chose to illustrate our data in the form of a heat map (Fig. 2). In the heat map, the levels of frailty in patients as determined by different frailty assessments (EFS, CFS, and Katz index) were compared by representing with specific colours (fit = green, vulnerable/ mildly frail = yellow, moderately frail = pink and severely frail = red). Frailty assessment in these patients showed that the median EFS of consented patients was 6 (IQR 5–8). The median CFS for the same consented patient population was 3 (IQR 3- 4) suggesting that most patients were managing well, despite their medical condition with a small proportion having CFS ≥ 5 (19.3%, data not shown). Conversely, the median Katz frailty index was 6 (IQR 6 -6), which showed that the majority of patients remained largely independent but 12.3% failed to show independence in at least one activity of daily living (i.e., Katz index < 6, data not shown). It was observed that when assessing frailty using standard tools which consider the different domains of frailty (EFS and CFS), most consented patients in our study population were vulnerable or had mild frailty.

**Table 1:** Baseline characteristics of patients who had consented to participate in the study

**Characteristic, n (%)**		**Consented** (*n* = 64)	
***Frailty measurements***	EFS	6	(5–8)
	CFS	3	(3–4)
	Katz	6	(6–6)
	Handgrip (L)	29.0	(20.7–32.3)
	Handgrip (R)	27.3	(21.4–34.1)
	Chair sit/ stand	4	(3–5)
***Demographic and risk factors***	Age, years	69	± 7
	Female sex	16	(25.0)
	BMI < 18.5 or ≥ 35 kg/m^2^	10	(15.6)
	Smoking history	46	(71.9)
	Diabetes	34	(53.1)
	Hypertension	55	(85.9)
	Dyslipidemia	56	(87.5)
	CVD	13	(20.3)
	PVD	14	(21.9)
	Renal failure	8	(12.5)
	COPD	11	(17.2)
	Pulmonary hypertension	2	(3.1)
***Heart function***	Atrial fibrillation	11	(17.2)
	MI ≤ 21 days	4	(6.2)
	LVEF < 40%	11	(17.2)
	CHF	13	(20.3)
	NYHA class 3–4	43	(67.2)
***Surgery***	Previous surgery	1	(1.6)
	Isolated CABG	51	(79.7)
	Isolated valve	6	(9.4)
	CABG + valve	7	(10.9)

*Abbreviations*: *BMI* Body Mass Index, *CVD* Cerebrovascular Disease, *PVD* Peripheral Vascular Disease, *COPD* Chronic Obstructive Pulmonary Disorder, *MI* Myocardial Infarction, *LVEF* Left Ventricle Ejection Fraction, *CHF* Congestive Heart Failure, *NYHA* New York Heart Association, *HCT* Hematocrit

### Patient exclusion

**Fig. 3:**
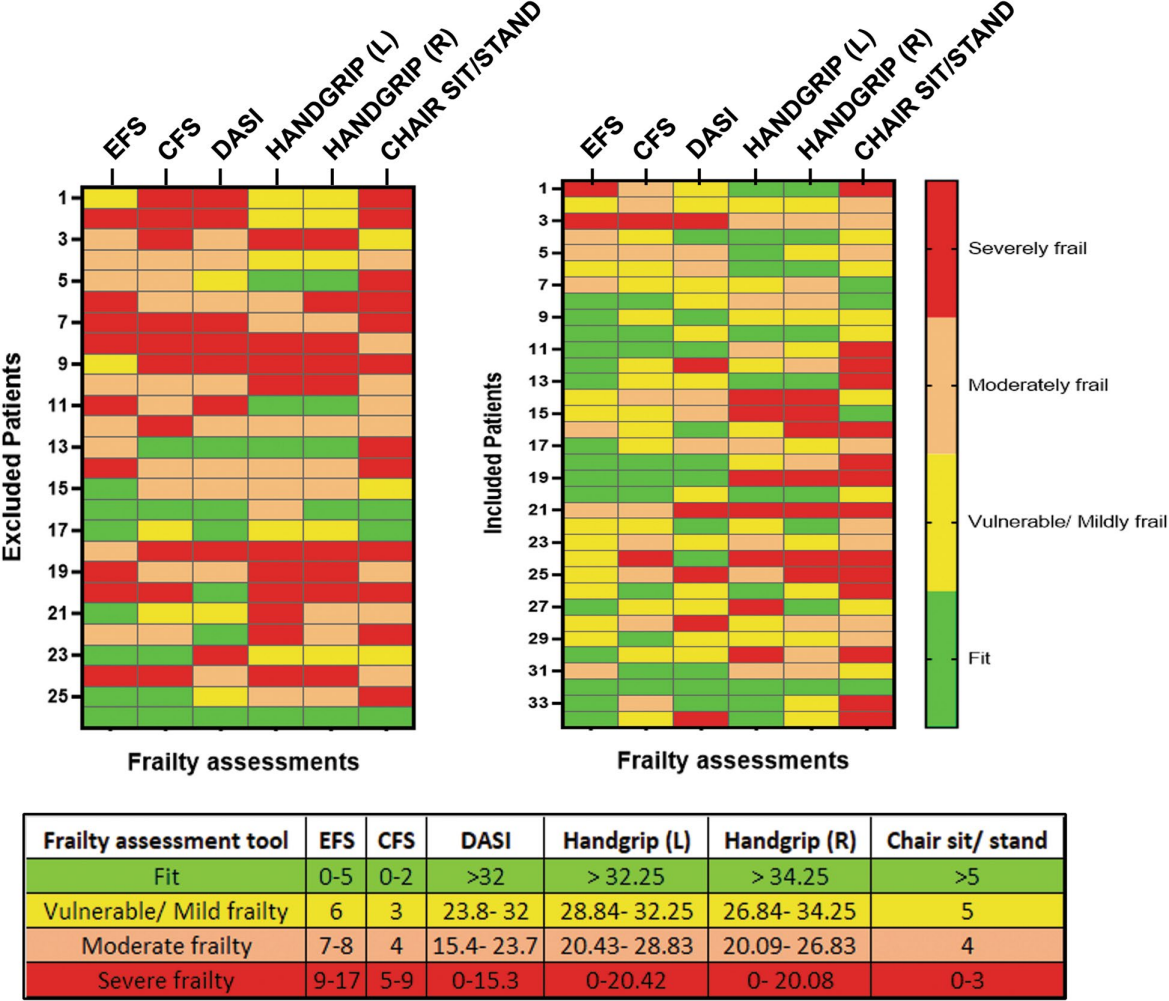
Heatmap for comparison of frailty between patients who were included in THE-FACS study versus those excluded. Patients were screened for frailty using different approaches- Edmonton Frailty Scale (EFS), Clinical Frailty Scale (CFS), Katz Index, Duke Activity Status Index DASI), Handgrip strength (left and right hand) and chair sit/ stand test. This frailty information was then compared in patients who were included in the study versus those who were excluded based on the inclusion and exclusion criteria (home discharge within 10 days of cardiac surgery). Frailty groups (fit, vulnerable/ mildly frail, moderately frail, and severely frail) were determined using the median and quartiles. The table shows the quartile ranges used in the individual frailty scores. Patients excluded from the study had a higher degree of frailty compared to those who were included in the study

Out of the 64 patients who consented to participate in THE-FACS, 29 patients (45.3%) needed to be excluded from THE-FACS program (Fig. 1) because of prolonged stay (LOS > 10 days) at the hospital (length of stay, LOS > 10 days, *n* = 15/61) or requiring hospital-to-hospital transfer to finish their recoverin in-hospital (12/61). Two other patients were excluded due to in-hospital mortality and inadequate literacy respectively (Fig. 1). Patients who were excluded from the study were frailer based on EFS and CFS (Table 2). The heat maps plotted for the different frailty assessments in these two groups of patients similarly showed that excluded patients appeared more frail with a greater proportion being moderate to severely frail (Fig. 3).

**Table 2:** Baseline characteristics and in-hospital outcomes of patients who were excluded versus who were included in the study

**Characteristic, n (%)**		**Excluded (*n* = 29)**		**Included (*n* = 35)**	***p* value**	
***Frailty measurements***	EFS	8	(5–10)	6	(5–6)	**0.003**
	CFS	4	(3–5)	3	(2–4)	**0.004**
	Katz	6	(6–6)	6	(6–6)	0.41
	Handgrip (L)	25.4	(19.2–31.3)	30.0	(27.0–35.0)	0.08
	Handgrip (R)	26.9	(20.2–33.2)	30.1	(23.3–34.7)	0.38
	Chair sit/ stand	4	(3–5)	4	(3–5)	0.84
***Demographic and risk factors***	Age, years	70	± 8	68	± 5	0.09
	Female sex	8	(27.6)	8	(22.9)	0.66
	BMI < 18.5 or ≥ 35 kg/m^2^	6	(20.7)	4	(11.4)	0.49
	Smoking history	20	(69.0)	26	(74.3)	0.64
	Diabetes	14	(48.3)	20	(57.1)	0.48
	Hypertension	23	(79.3)	32	(91.4)	0.28
	Dyslipidemia	24	(82.8)	32	(91.4)	0.45
	CVD	10	(34.5)	3	(8.6)	**0.01**
	PVD	6	(20.7)	8	(22.9)	0.83
	Renal failure	5	(17.2)	3	(8.6)	0.45
	COPD	6	(20.7)	5	(14.3)	0.53
	Pulmonary hypertension	1	(3.4)	1	(2.9)	1.0
***Heart function***	Atrial fibrillation	7	(24.1)	4	(11.4)	0.20
	MI ≤ 21 days	3	(10.3)	1	(2.9)	0.32
	LVEF < 40%	8	(27.6)	3	(8.6)	**0.05**
	CHF	9	(31.0)	4	(11.4)	**0.05**
	NYHA class 3–4	19	(65.5)	24	(68.6)	0.80
***Surgery***	Previous CV	9	(31.0)	7	(20.0)	0.31
	Previous surgery	1	(3.4)	0	(0.0)	0.45
	Urgent status	9	(31.0)	14	(40.0)	0.46
	HCT < 24%	0	(0.0)	0	(0.0)	-
	Procedure					
	CABG	22	(75.9)	29	(82.9)	0.35
	Valve	2	(6.9)	4	(11.4)	
	CABG + valve	5	(17.2)	2	(5.7)	
***In-hospital outcomes***	LOS	11	(7–12)	5	(5–7)	** < 0.001**
	Discharge disposition					
	Home	13	(44.8)	35	(100)	** < 0.001**
	Hospital	15	(51.7)	0	(0.0)	
	Expired	1	(3.4)	0	(0.0)	

*Abbreviations*: *BMI* Body Mass Index, *CVD* Cerebrovascular Disease, *PVD* Peripheral Vascular Disease, *COPD* Chronic Obstructive Pulmonary Disorder, *MI* Myocardial Infarction, *LVEF* Left Ventricle Ejection Fraction, *CHF* Congestive Heart Failure, *NYHA* New York Heart Association, *HCT* Hematocrit

In the remaining 35 patients, who were discharged home with THE-FACS, more than 1/3 failed to complete the study (n = 14 patients withdrew, 40%, Fig. 1). This was largely due to technical difficulties during the intervention (10/14) including issues with the software on the tablet, inability to use technology, lack of prior exposure to modern technology, or difficulties in getting help from family/ caregivers for completing the daily health checks.

### Final patient cohort reaching study completion

The remaining 21 patients able to complete the study transmitted the Healthchecks daily for the 30-day study period (i.e., compliance rate was 100%). During the study period, there was a total of 10 alarm cases and subsequent 10 phone calls that were made to 9 participants out of the 21 participants who completed the study. These 21 patients were compared to 2:1 propensity score-matched historical controls (*n* = 42) (Fig. 1). The groups were well matched in demographic characteristics (completed and control, Table 3). When comparing 30-day outcomes, patients who completed THE-FACS program had reduced rates of hospital re-admission (0% versus 14.3%, *p* = 0.17) and any type of infection (9.5% versus 21.4%, 0.31) compared to historical controls but this failed to reach significance (Table 4).

**Table 3:** Comparison of baseline characteristics patients who completed the study versus twice the number of propensity score-matched historical controls

**Characteristic, n (%)**		**Control (*n* = 42)**		**Completed (*n* = 21)**		***p*-value**
***Demographic and risk factors***	Age, years	66	± 7	68	± 5	0.29
	Female sex	2	(4.8)	4	(19.0)	0.09
	BMI < 18.5 or ≥ 35 kg/m^2^	7	(16.7)	3	(14.3)	1.00
	Smoking history	29	(69.0)	15	(71.4)	0.85
	Diabetes	20	(47.6)	12	(57.1)	0.48
	Hypertension	40	(95.2)	19	(90.5)	0.60
	Dyslipidemia	42	(100.0)	21	(100.0)	-
	CVD	2	(4.8)	1	(4.8)	1.00
	PVD	11	(26.2)	5	(23.8)	0.84
	Renal failure	1	(2.4)	1	(4.8)	1.00
	COPD	8	(19.0)	3	(14.3)	0.74
	Pulmonary hypertension	0	(0.0)	0	(0.0)	-
***Heart function***	Atrial fibrillation	9	(21.4)	3	(14.3)	0.74
	MI ≤ 21 days	3	(7.1)	1	(4.8)	1.00
	LVEF < 40%	0	(0.0)	0	(0.0)	-
	CHF	0	(0.0)	0	(0.0)	-
	NYHA class 3–4	31	(73.8)	15	(71.4)	1.00
***Surgery***	Previous CV	7	(16.7)	3	(14.3)	1.00
	Previous surgery	2	(4.8)	0	(0.0)	0.55
	Urgent status	22	(52.4)	9	(42.9)	0.48
	HCT < 24%	0	(0.0)	0	(0.0)	-
	Procedure					
	CABG	37	(88.1)	17	(81.0)	0.72
	Valve	3	(7.1)	3	(14.3)	
	CABG + valve	2	(4.8)	1	(4.8)	

*Abbreviations*: *BMI* Body Mass Index, *CVD* Cerebrovascular Disease, *PVD* Peripheral Vascular Disease, *COPD* Chronic Obstructive Pulmonary Disorder, *MI* Myocardial Infarction, *LVEF* Left Ventricle Ejection Fraction, *CHF* Congestive Heart Failure, *NYHA* New York Heart Association, *HCT* Hematocrit

**Table 4:** Comparison of 30-day outcomes between patients who completed the study versus twice the number of propensity score-matched historical controls

**Outcome, n (%)**	**Control**		**Completed**		***p*-value**
	***n* = 42**		***n* = 21**		
**30-day**					
**Mortality**	0	(0.0)	0	(0.0)	-
**Readmission**	**6**	**(14.3)**	**0**	**(0.0)**	**0.17**
**Any infection**	9	**(21.4)**	**2**	**(9.5)**	**0.31**
**Renal failure**	3	(7.1)	3	(14.3)	0.39
**Delirium**	2	(4.8)	3	(14.3)	0.32
**CVA/ TIA**	1	(2.4)	0	(0.0)	1.00
**Atrial fibrillation**	25	(59.5)	11	(52.4)	0.59

*Abbreviations*: *MI* Myocardial Infarction, *CVA* Cerebro Vascular Accident, *TIA* Transient Ischemic Attack

### Using THE-FACS intervention yielded high participant satisfaction

**Fig. 4:**
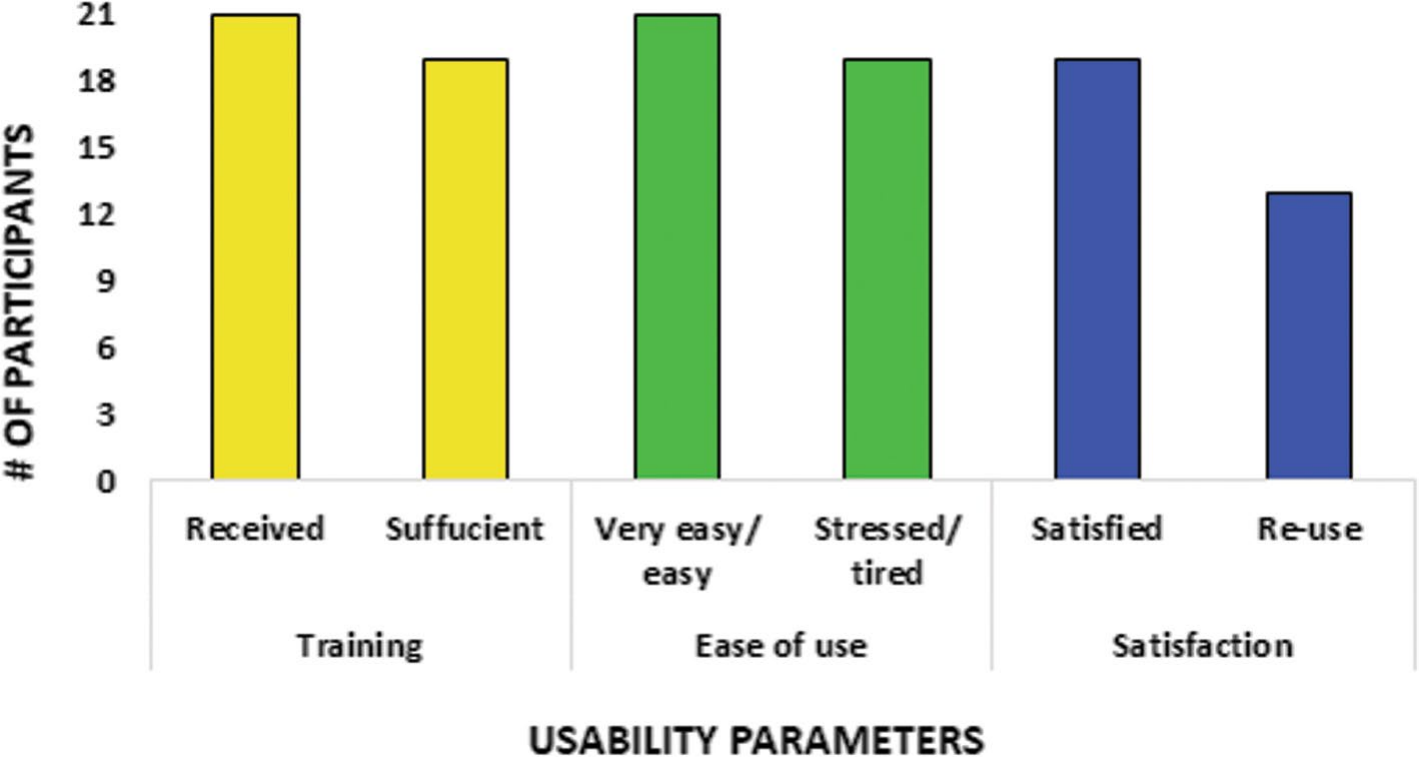
Participant satisfaction survey. A satisfaction survey was conducted to understand the opinion of the participants who completed THE-FACS study. Most patients felt that the intervention was easy to use and not stressful. Patients were also highly satisfied with THE-FACS and would be willing to use another home monitoring intervention in the future

A satisfaction survey to obtain participant feedback who completed the study revealed that THE-FACS was readily accepted and provided a high level of patient satisfaction. Over 90% of patients showed a strong agreement for all questions. Specifically, 21/21 (100%) patients found THE-FACS very easy/easy, 20/21 (95.2%) were not stressed/tired during their monitoring, 20/21 (95.2%) were satisfied and 14/21 (66.7%) would like to use a home monitoring device again (Fig. 4).

## Discussion

Advances in technological interventions to remotely monitor patients after cardiac surgery are increasingly being explored to improve outcomes [28]. As such we designed a pilot study to test the feasibility of using a novel telehealth intervention to particularly target vulnerable or frail patients undergoing heart surgery. We believed that our study would address the important knowledge gap by assessing if technological interventions could help patients transition home after discharge. Our study demonstrated that a large proportion of patients undergoing cardiac surgery were vulnerable and eligible to participate (34%) highlighting the need for our work. We were also able to enroll a large proportion of patients (74%) but did encounter some challenges as only 1/3 of enrolled patients completed the study as designed.

Our results demonstrated that although it was possible to enroll and remotely monitor patients following cardiac surgery, frail/ vulnerable patients pose unique challenges for this type of intervention. Specifically, more than half of our consented patients had to be excluded from our study because they could not be discharged home within 10 days of surgery or required transfer to another hospital for recovery before they could be discharged home. Most of these excluded patients had a much higher frailty score (as defined by EFS, CFS) than the ones who were included in the study. While not surprising, these observations highlight how progressively frail patients have an increased burden of comorbidities and suffer a greater number of adverse postoperative outcomes and slower overall recovery [29, 30]. Our findings are supported by previous reports observed in frail patients undergoing cardiac surgery in other Canadian populations [31–33]. We acknowledge that we chose to exclude patients that needed prolonged hospitalization given that the goal of the telehealth intervention was to facilitate early transition home after discharge. It also does not mean that the patients that were excluded due to prolonged hospitalization might not have benefited from the technology.

It is also important to note that the actual tablet platform did have some issues that are worth reporting. One-third of the participants sent home with the tablet device withdrew from our study, predominantly due to difficulties in using the technology. Technology, especially for older adults is a complex relationship encompassing psychological and contextual factors [34] with perceived barriers not limited to lack of prior knowledge, fear of consequences, and lack of readiness in using a technological intervention [35]. Many of the patients who withdrew had the above perceptional barriers and could not avail assistance from family or friends to help them with using the tablet and the blood pressure device. Additionally, our observations suggest that self-monitoring every day at a fixed time for 30 consecutive days may also be a reason for stress/ monotony/ fatigue and the consequent withdrawals. Despite these barriers, among patients who completed the study, there was a high degree of satisfaction and willingness to again use technology-assisted programs to monitor their health.

We chose hospital readmission as the primary clinical outcome of interest for THE-FACS intervention. This was based on the available literature, and unpublished data from the NBHC, showing that frail patients have a higher risk of hospital readmission within 30 days of cardiac surgery [32, 36]. It is generally reported that rates of readmission after cardiac surgery can range between 10–15% and are influenced largely by patient characteristics [37]. In the present pilot study, none of the patients who received THE-FACS intervention required readmission to the hospital. This finding was in contrast with our historical controls which had a rate of readmission of 14.3%. However, one should note that despite this apparent difference between THE-FACS intervention and historical controls, the results failed to reach statistical significance. This is not surprising given the small size of our pilot study which was not powered to detect a difference between groups. Given that this was a pilot study, it was not possible to prospectively enroll controls or have randomization. We acknowledge this limitation in the present study. Furthermore, the proportion of females in the control group was lower compared to THE-FACS intervention group, and any confounding factors based on sex cannot be ruled out. Our observations are interesting and suggest that a telehealth intervention could have a significant impact on facilitating discharge home but will need to be validated in a larger cohort. It remains to be proven as to the utility of technology-based home monitoring programs to reduce the rates of hospital readmissions in vulnerable/ frail patients within 30 days of cardiac surgery.

Most patients who completed the study were highly satisfied with THE-FACS intervention and most would like to use the technology again for home monitoring of their health. However, the satisfaction survey was not conducted on patients who withdrew consent and did not complete the study and is as such a limitation of our data. As the maximum number of participants in the latter group faced technological difficulties when using the intervention, we acknowledge that our satisfaction survey would have been biased and reflected on only those participants who are technologically proficient to complete the study.

We, therefore, conclude that THE-FACS is a telehealth intervention that holds promise in our quest to identify solutions for increasing vulnerable or frail patients undergoing surgery who need help with their transition to home life. Our findings suggest that perhaps this type of intervention could help reduce hospital readmission within 30 days of surgery but would require a much larger study to answer that question. However, we also identified significant challenges in applying this approach to the most vulnerable or frail patients suggesting that unique solutions would need to be developed to limit withdrawal or dismissal of the technology. One needs to keep in mind that the most vulnerable patients were the most likely to require prolonged hospitalization and as such were excluded from the present pilot.

Although we observed how novel, self-monitoring technological interventions can prove to be challenging for the most frail/ vulnerable, elderly patients who had undergone cardiac surgery, we could see its tremendous potential if adapted to individual needs. A hybrid approach to monitoring health and recovery post- surgery, using technology and traditional methods according to the needs of the patients is perhaps a better method of patient management that remains to be proven. As a continuation of the potential benefits of home-monitoring previously mentioned [4], it would be important to explore the applicability of digital therapeutics in a larger cohort of patients undergoing cardiac surgery in New Brunswick. A risk/ benefit analysis considering multiple aspects not limited to outcomes, costs, time, resources, etc. for both the patients and their caregivers/ family, and the healthcare system would provide a comprehensive knowledge regarding the large-scale utility and long-term sustainability of technology-based home monitoring platforms in cardiac surgery patients.

## Supplementary Information

**Additional file 1**: **Supplementary Figure 1.** Schematic diagram of THE-FACS study Schematic diagram showing the steps involved in the study.

**Additional file 2**: **Supplementary Table 1**. Usability survey for THE-FACS intervention using Likert-like questions.

## Data Availability

All data generated or analyzed during this study are included in this published article (and its supplementary information files).

## Abbreviations

BMI: Body Mass Index
CABG: Coronary Artery Bypass Graft
CFS: Clinical Frailty Scale
CFN: Canadian Frailty Network
CHF: Congestive Heart Failure
COPD: Chronic Obstructive Pulmonary Disorder
CVA: Cerebro Vascular Accident
CVD: Cerebrovascular Disease
DASI: Duke Activity Status Index
EFS: Edmonton Frailty Scale
HCT: Hematocrit
IQR: Inter Quartile Range
LOS: Length Of Stay
LVEF: Left Ventricle Ejection Fraction
MI: Myocardial Infarction
NBHC: New Brunswick Heart Centre
NBHRF: New Brunswick Health Research Foundation
NYHA: New York Heart Association
PVD: Peripheral Vascular Disease
REB: Research Ethics Board
THE-FACS: Telehealth Home monitoring Enhanced-Frailty And Cardiac Surgery
TIA: Transient Ischemic Attack
