# Supplementary figures and images for: Enhanced Telehealth Home-Monitoring Intervention for Vulnerable and Frail Patients after Cardiac Surgery (THE-FACS Pilot Intervention Study)
Source: BMC Geriatr. 2022 Nov 5;22:836. doi: 10.1186/s12877-022-03531-4 (PMC9636804; doi:10.1186/s12877-022-03531-4)

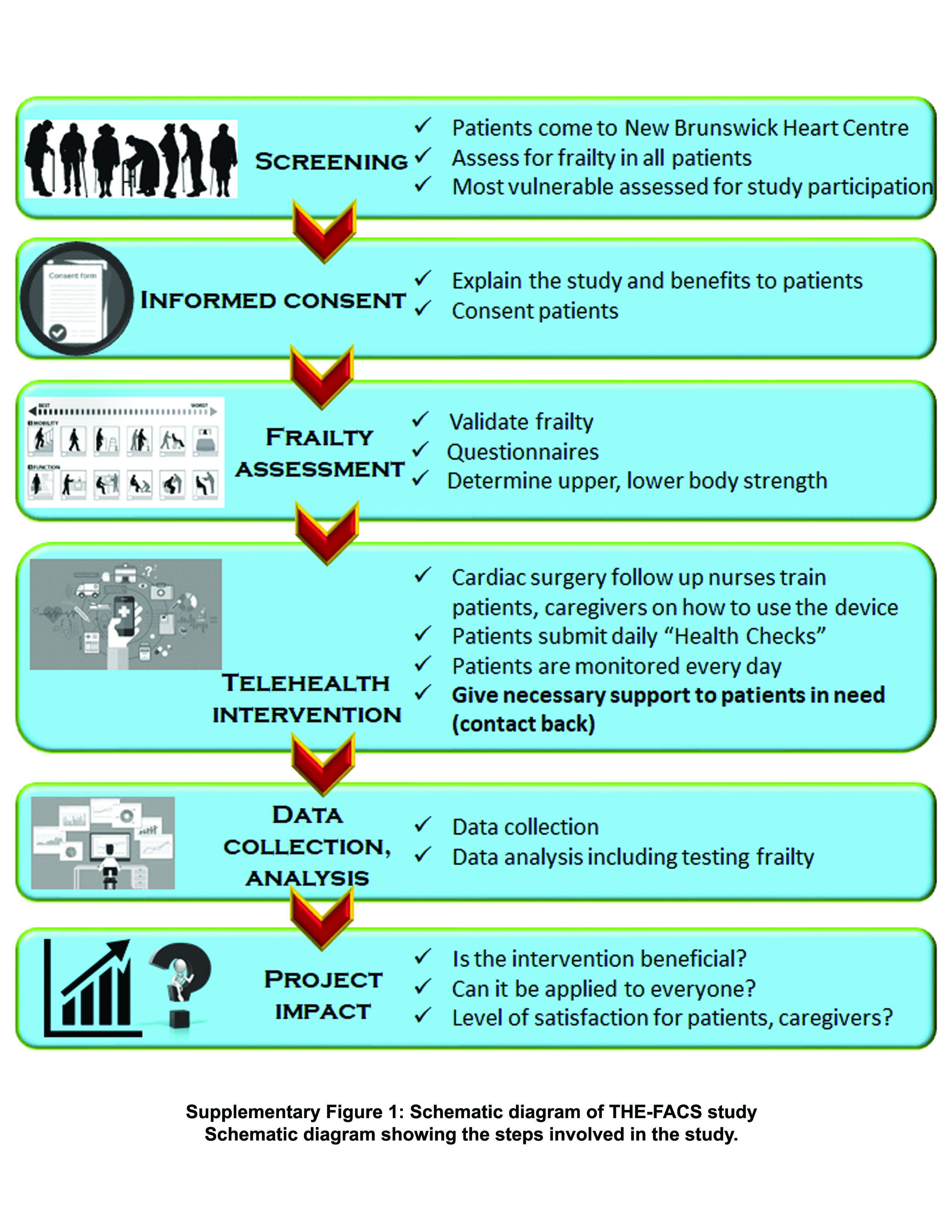

Supplement: Supplementary file 1 — Additional file 1: Supplementary Figure 1. Schematic diagram of THE-FACS study Schematic diagram showing the steps involved in the study. [file 12877_2022_3531_MOESM1_ESM.tif]
