# Supplementary material for: Enhanced Telehealth Home-Monitoring Intervention for Vulnerable and Frail Patients after Cardiac Surgery (THE-FACS Pilot Intervention Study)
Source: BMC Geriatr. 2022 Nov 5;22:836. doi: 10.1186/s12877-022-03531-4 (PMC9636804; doi:10.1186/s12877-022-03531-4)
Supplement: Supplementary file 2 — Additional file 2: Supplementary Table 1. Usability survey for THE-FACS intervention using Likert-like questions. [file 12877_2022_3531_MOESM2_ESM.docx]

|  |  | **PERSON COMPLETING THE FORM:**  **□ PATIENT □ OTHER** | |  |  | **Date:** ____________________ | |
| --- | --- | --- | --- | --- | --- | --- | --- |
|  |  |  | Strongly disagree | Disagree | Neutral | Agree | Strongly agree |
| 1 | **Training** | Were you given training on how to use the monitoring device? |  |  |  |  |  |
| 2 |  | Were you comfortable with the amount of training to operate the device you received? |  |  |  |  |  |
| 3 | **Ease of use** | Was it easy to complete your health checks? |  |  |  |  |  |
| 4 |  | Was it tiring or stressful to use the device? |  |  |  |  |  |
| 5 | **Satisfaction** | Were you happy to use the monitor to do health checks |  |  |  |  |  |
| 6 |  | If given a chance, would you like to use a home monitoring device again to monitor your health? |  |  |  |  |  |

**Supplementary Table 1**: Usability survey for THE-FACS intervention using Likert-like questions.
